# Integrating large language models in mental health practice: a qualitative descriptive study based on expert interviews

**DOI:** 10.3389/fpubh.2024.1475867

**Published:** 2024-11-04

**Authors:** Yingzhuo Ma, Yi Zeng, Tong Liu, Ruoshan Sun, Mingzhao Xiao, Jun Wang

**Affiliations:** Department of Nursing, The First Affiliated Hospital of Chongqing Medical University, Chongqing, China

**Keywords:** large language model, qualitative study, ChatGPT, public health, mental health

## Abstract

**Background:**

Progress in developing artificial intelligence (AI) products represented by large language models (LLMs) such as OpenAI’s ChatGPT has sparked enthusiasm for their potential use in mental health practice. However, the perspectives on the integration of LLMs within mental health practice remain an underreported topic. Therefore, this study aimed to explore how mental health and AI experts conceptualize LLMs and perceive the use of integrating LLMs into mental health practice.

**Method:**

In February–April 2024, online semi-structured interviews were conducted with 21 experts (12 psychiatrists, 7 mental health nurses, 2 researchers in medical artificial intelligence) from four provinces in China, using snowballing and purposive selection sampling. Respondents’ discussions about their perspectives and expectations of integrating LLMs in mental health were analyzed with conventional content analysis.

**Results:**

Four themes and eleven sub-themes emerged from this study. Firstly, participants discussed the (1) practice and application reform brought by LLMs into mental health (fair access to mental health services, enhancement of patient participation, improvement in work efficiency and quality), and then analyzed the (2) technological-mental health gap (misleading information, lack of professional nuance and depth, user risk). Based on these points, they provided a range of (3) prerequisites for the integration of LLMs in mental health (training and competence, guidelines for use and management, patient engagement and transparency) and expressed their (4) expectations for future developments (reasonable allocation of workload, upgrades and revamps of LLMs).

**Conclusion:**

These findings provide valuable insights into integrating LLMs within mental health practice, offering critical guidance for institutions to effectively implement, manage, and optimize these tools, thereby enhancing the quality and accessibility of mental health services.

## 1 Introduction

Mental health is a primary component of modern public health. With the rapid development of digital tools in the mental health field, various artificial intelligence (AI) products are increasingly being developed to support mental health professionals in managing time-consuming and repetitive tasks (1). Moreover, AI-virtual and robotic agents are not only utilized for relatively low-level tasks, such as case analysis or health education, but also at high-level clinical work, including treatment solutions for mental health care (2). Recently, progress in developing AI products represented by large language models (LLMs) such as ChatGPT, Google Bard, and Bing-Chat has sparked enthusiasm for their potential use in mental health care (3). LLMs are based on deep neural networks trained on vast amounts of data from online sources, including web pages, books, and social media, to generate human-like text (4). Their conversational interactivity and equal-to-human-level performance in cognitive tasks across fields, including mental health care, have created unprecedented opportunities for analyzing and generating language data on a massive scale (5). Related research shows that LLMs have the potential to transform mental health practice because language data has a central role in this area (3).

The potential use of AI products in mental health practice has often been controversial, and LLMs are no exception. On the one hand, numerous studies have emphasized that LLMs could be used to (a) provide mental health counseling, (b) identify specific mental conditions, (c) predict and diagnose mental illness (6, 7). As with many new technologies, LLMs in mental health practice are a double-edged sword. The application of LLMs faces significant challenges, primarily due to the inherent limitations of LLMs and improper use by practitioners. Firstly, these tools may generate misleading information due to biases in the training data, which is particularly dangerous when dealing with sensitive data on human behavior and emotions in mental illness (8). Secondly, the lack of interpretability in these models makes it difficult for mental health professionals to understand the reasoning processes, affecting the transparency and reliability of clinical decisions (9). Additionally, unreasonable use of these tools could compromise patient privacy or safety. Currently, these issues remain persistent and are highly controversial in ethical discussions of mental health (10). Therefore, some scholars argue LLMs are not yet ready to provide mental health assessments and interventions (11).

By reviewing current published peer-reviewed papers on the use of LLMs in mental health, we can discover that most papers consist only of reviews, commentaries, or editorials to discuss the potential and challenges of applying LLMs to mental health (12). Moreover, some interventional research may not have gone through a formal review process. A previous empirical study – one qualitative interview study – has focused on exploring the views of patients concerning the efficacy and potential negative impacts of using ChatGPT for mental health support in an outpatient setting (13). Regrettably, the study did not incorporate experts’ perspectives, as these could provide additional insights into clinical applicability and ethical considerations of such technology. Additionally, experts’ viewpoints could help determine whether—and on what timescale—LLMs should be developed and disseminated.

This study aimed to fill the gap and explore how mental health and AI experts conceptualize LLMs and perceive the use of integrating LLMs into mental health practice. In addition to gaining insight into the experts’ attitudes, our goal was to analyze their perspectives and expectations in connection with the broader social context of LLM development. In this article, we described experts’ views concerning the reforms and challenges brought by LLMs, and then summarized the prerequisites and conditions for LLMs integration. Eventually, we formulated concrete recommendations to support successful implementation of LLMs in mental health practice. This contributes to mental health professionals harnessing their power for research while avoiding the harm that might come from premature applications.

## 2 Methods

### 2.1 Study design setting

This was a qualitative descriptive study conducted from February to April 2024 using online semi-structured interviews. The qualitative descriptive design was chosen because it enabled an understanding of experts’ perceptions and expectations regarding integrating LLMs in mental health practice in China, an important topic sparsely documented in the literature. This approach thereby contributes to the body of knowledge as well as to the implementation and management of these tools in mental health practice and the future development of the technology. Additionally, the qualitative descriptive design is a distinctive component of qualitative research that is valuable especially when direct descriptions of phenomena including who, what, and where are desired by the researcher. This study directly described the mental health and AI experts’ perspectives on the application of LLMs, facilitating the development of comprehensive evidence-based knowledge. The study design was guided by the consolidated criteria for reporting qualitative studies (COREQ) (14) (Supplementary material S1).

### 2.2 Sampling and recruitment

In February–April 2024, purposive and snowball sampling methods were utilized to recruit participants who represented a diverse range of characteristics, including mental health-related profession, age, gender, and education level (12 psychiatrists, 7 mental health nurses, 2 researchers in medical AI). Two mental health nurses from our research team sent a recruitment email describing the details of the study (purpose, interview process, and analysis plan) to professional researchers. Then we invited the respondents who were interested in the interviews to complete an online questionnaire including their basic information, more specific research content, and some items regarding the understanding of LLMs. We comprehensively considered the experts’ general information through group meetings, including years of work experience, professional titles, publication records in the past 5 years, and their frequency of use and understanding of LLMs. Those who met the criteria received another email with an informed consent form. Upon confirmation of participation, specific arrangements were made for the online video interviews. Out of the 40 responses received, 8 respondents declined to participate, and 11 respondents did not meet the inclusion criteria. The rest of the interviewees completed the interview successfully. The inclusion criteria: (a) voluntarily respond to the invitation for study participation; (b) have experience in AI or mental health-related work over 10 years; (c) be familiar with LLMs; (d) can speak and understand Chinese, as the study activities are conducted in China; (e) the frequency of using LLMs was categorized as “often” or “always”; (f) at least 3 SCI papers published in the past 5 years; (g) hold an intermediate or senior professional title. The exclusion criteria: (a) participants who had resigned; (b) have any medical or psychological conditions that might interfere with their ability to participate in the study.

### 2.3 Data collection and interview process

Individuals who met the inclusion criteria were invited to an online individual semi-structured interview using the most popular remote conferencing platform in China (Tencent Meeting) at their preferred time. The interview guide was developed on the team’s preliminary scoping review and bibliometric analysis: The Application of ChatGPT in Medicine: A Scoping Review and Bibliometric Analysis (15), and followed the 5-step process of qualitative semi-structured interview guide presented in a systematic methodological review (16). We pilot-tested the interview guide with two participants to identify and remedy any problems. As we progressed in our data collection, the interview guide was modified to adapt interviews (Supplementary material S2). The first author, a Master of Medicine specializing in qualitative research, acted as the moderator during the online interviews. Additionally, a co-author, a mental health nurse, assisted in scheduling an online meeting time and recorded field notes on other signals (tone, speech rate, and emotion). Each interview, lasting approximately 30 to 60 min, was transcribed and then coded after its conclusion. Additionally, the first and second authors constructed a saturation coding table to record the number of codes, themes, shared themes with previous interviews, new themes per interview, and the total number of themes. The content of each interview was then compared with the previous ones to track any new or recurring themes. During the 19th interview few new codes were generated and the findings largely overlapped, indicating the information had reached saturation (17). Two additional interviews were conducted to confirm the saturation. Because no new data emerged in the last three interviews, data saturation was confirmed, and no new participants were sought. Due to the sensitivity of the subject matter, participants provided their responses with the video function disabled to ensure the authenticity of their perspectives.

### 2.4 Data management and analysis

The Tencent Conference Application was used for recording and initial transcription. A female researcher with a master’s degree checked the transcripts for accuracy and corrected these as necessary, adding the emotions and notes during transcription to reflect the meaning of the words.

The comprehensive analysis included: (a) two researchers repeatedly reviewed the interview materials and immersed themselves in the data to achieve a comprehensive understanding of the content; (b) they annotated significant content and key information within the materials; (c) they coded the first three transcripts independently to compare coding consistency using Nvivo12. In cases of disagreement, the broader research team was consulted to reach consensus themes, after these three interviews, we continued with consensus discussions to ensure the consistency and comprehensiveness of the coding; (d) reflections on professional researchers’ perspectives of integrating LLMs into mental health practice were labeled and categorized and the coding was compared and discussed to form the coding sheets; (e) similar and relevant codes were categorized to form themes and subthemes; (f) corresponding excerpts were selected from the materials as examples.

### 2.5 Rigour and reflexivity

To ensure rigour in analysis, we established credibility, transferability, dependability, and confirmability. Credibility and dependability were achieved through regularly scheduled group discussion sessions, comprehensive reviews of the collected data, and member checks, wherein participants were allowed to review and edit their responses following the interview. The transferability of this study was ensured by providing rich, detailed descriptions of the study participants and the research context (psychiatrists, mental health nurses, researchers in medical AI from different provinces in China), allowing others to determine the applicability of the findings to similar settings. Confirmability was achieved through maintaining an audit trail and triangulation of data sources.

A reflexive journal was kept by the first author in the form of a paper diary and meeting minutes used to track interview scheduling and document written reflections and memos throughout the research process. The majority of the authors had extensive experience and knowledge in conducting research into mental health and qualitative research. To ensure a true reflection of the data, they introspected on the pre-existing knowledge, biases, and assumptions during the interview, analysis, and write-up process continuously.

### 2.6 Ethics

This study was approved by the ethical committee of First Affiliated Hospital of Chongqing Medical University, file 2023–334. All participants were fully informed about the purpose of the study and provided consent before conducting the semi-structured interviews. For confidentiality, all interview data, related descriptions, and record files were stored on the hard drive of a password-protected computer shared by only the authors; backup files were secured in locked file cabinets.

## 3 Results

### 3.1 Study characteristics

In February–April 2024, semi-structured interviews were conducted with 21 experts from four provinces in China. The duration of each interview ranged from 30 to 60 min. The participants were a mixture of experienced mental health experts and professionals with an active role in digitalization or AI development, including 12 psychiatrists, 7 mental health nurses, and 2 researchers in the field of AI and medicine. 12 were female and 9 were male, and ages ranged from 22 to 60 years old. Characteristics of the participants are shown in Table 1.

**Table 1:** Characteristics of the participants.

Characteristic	Participants, *n* (%)
Age	
<30	3 (14.29%)
30–40	8 (38.10%)
>40	10(47.62%)
Occupation	
Psychiatrists	12 (57.14%)
Mental health nurse	7 (33.33%)
Researchers in Medical Artificial Intelligence	2 (9.52%)
Sex	
Male	9 (42.86%)
Female	12 (57.14%)
Educational background	
Bachelor	3 (14.29%)
Master	7 (33.33%)
PhD	11 (52.38%)
Experience working in the field	
10–15 years	6 (28.57%)
More15years	13 (61.90%)
Unknown	2 (9.52%)

### 3.2 Major themes

Comments were classified into four themes and 11 sub-themes: (1) practice and application reform in mental health (fair access to mental health services, enhancement of patient participation, improvement in work efficiency and quality), (2) technological-mental health gap (misleading information, lack of professional nuance and depth, user risk), (3) prerequisites for the integration of LLMs (training and competence, guidelines for use and management, patient engagement and transparency), (4) expectation and future of LLMs in mental health (reasonable allocation of workload, upgrades and revamps of LLMs). The codes and themes from the analysis were organized in Table 2.

**Table 2:** The process of topic extraction.

Theme	Sub-Theme	Quote
Practice and application reform in mental health	Promote equity in public access to mental health services	(…) medical LLMs could be deployed through mobile apps or online platforms, making related psychological advice and support accessible to patients in remote or underserved regions who have internet access.
	Facilitate patient engagement in mental health therapy	As a virtual assistant, LLMs could provide patients with a responsive, always-available channel for communication that can overcome barriers to seeking psychological counseling and encourage them to engage in therapeutic practices.
	Improve the efficiency and quality of work	(…) It can help me handle some basic repetitive tasks efficiently. (…) LLMs can reduce the administrative burden on mental health professionals and allow them more time to focus on patient care. (…) By analyzing vast amounts of medical literature and patient data, these models can provide real-time decision support.
technological- mental health gap	Generation of misleading information	If the training data contains inaccuracies or is not comprehensive enough to cover all scenarios and nuances, LLMs may generate misleading information based on that flawed data.
	Lack of professional nuance and depth	LLMs are designed to generate text based on what they have learned from training rather than deep clinical understanding. (…) The generated content is too generic to act as a professional.
	User risk	The behavior of psychiatrists and mental health nurses in their use of LLMs poses significant potential risks which include reliance, sensitive patient data, and ethical standards…
prerequisites for LLMs integration in mental health	Training and Competence	Currently, some mental health professionals around me have not even heard of LLMs, it is necessary to raise their awareness. (…) Digital literacy has become the core competitiveness of medical staff.
	Guidelines for Use and Management	The lack of research on the application of LLMs in mental health practice is largely due to the absence of relevant usage standards and management protocols.
	Patient Engagement and Transparency	Patients have the right to be informed that we are using LLMs to provide services for them
Expectations and future of LLMs in mental health	Reasonable allocation of workload	Healthcare organizations might expect clinicians to handle more patients or tasks in a given time frame due to the efficiencies gained from using LLMs, this is very dangerous.
	Upgrades and revamps of LLMs	LLMs should be continuously updated and upgraded to provide more personalized content, protect user information, and be designed more reasonably…

#### 3.2.1 Practice and application reform in mental health

##### 3.2.1.1 Fair access to mental health services

Relevant experts expressed varying degrees of value about how LLMs might provide mental health services. They emphasized that with the rapid developments in society, an increasing number of people would face mental health issues (N7). However, there might be a severe shortage of psychiatrists, mental health nurses, and other mental health resources, resulting in a significant gap in services. The gap not only limited patients’ access to necessary psychological support but also intensified the strain on public health systems. Therefore, urgent measures were needed, and LLMs were a good choice that could promote equity in public access to psychological services.

*“… LLMs have the potential to offer psychological counseling through online platforms, this is particularly beneficial for those in remote or people who may not have the opportunity to visit a professional” N4.*

*“The cost of psychological counseling services can be prohibitively expensive. If LLMs can perform preliminary evaluations for the public, it will undoubtedly alleviate the financial burden on the public” N8.*

They highlighted the equity in public access was primarily because of the ease of accessing certain LLMs, which were not restricted by location or time. Additionally, the lower cost associated with these models also encouraged patients to try these services.

##### 3.2.1.2 Enhancement of patient participation

If equity in public access to mental health services represents the initial crucial step, then ensuring consistent patient engagement throughout the therapy process is the essential subsequent phase.

*“LLMs can provide continuous emotional support and psychological health advice to patients in a conversational form, through the feedback mechanism, it can stimulate patients to participate and engage in their therapy” N21.*

Additionally, some participants mentioned digital platforms for psychological support had indeed been shown to reduce barriers for those who might feel stigmatized or uncomfortable seeking help face-to-face.

*“…like schizophrenia, patients or their families prefer seeking advice online rather than directly discussing the condition with us, the emergence of LLMs can help resolve this issue” N18.*

Based on the advantages mentioned above, patients’ behaviors of engaging in therapy for mental health problems enhance the personalization and specificity of therapeutic interventions, making treatment plans more closely to their needs. Eventually, it improves the outcomes of their conditions and increases patients’ trust and satisfaction with the therapeutic process.

##### 3.2.1.3 Improvement in work efficiency and quality

Experts also emphasized the importance of LLMs for psychiatrists and mental health nurses, primarily reflected in efficiency and quality of work. As virtual assistants, LLMs could assist with routine text-generation tasks, which allowed psychiatrists to devote more time and attention to patient care, leading to high-quality psychological services.

*“…Besides clinical work, psychiatrists have responsibilities such as writing reports, creating informative and educational content for the public, and performing other duties. LLMs can alleviate these burdens and free up more time for them to focus on patient care” N20.*

While LLMs enhanced efficiency and quality primarily in routine text-processing tasks and might not be suitable for specialized tasks such as mental health decision-making, some experts reported that future developments would enable LLMs to address professional tasks, thereby improving the efficiency and quality of clinical work.

*“In the future, by analyzing vast amounts of textual data through advanced computational methods, these models may be able to identify complex psychological conditions difficult to detect by a psychiatrists or mental health nurse alone” N9.*

*“LLMs have the potential to provide diagnostic support based on the data, and interventions informed by a wide array of existing mental health research and outcomes. The entire process enhances the accuracy and efficiency of psychological therapy and constructs personalized treatment plans tailored to individual needs” N12.*

#### 3.2.2 Technological-mental health gap

##### 3.2.2.1 Misleading information

Most experts reported that one of the key factors limiting the application of LLMs in mental health was their inherent limitations, which led to the potential dissemination of misleading information. These models operated by analyzing patterns in large datasets, rather than truly understanding the content, which could compromise their reliability in specialized fields such as mental health (N11).

*“Lots of literature points out that LLMs generated medical-related information with biases and hallucinations, so, there is a possibility of misdiagnosis of mental illness.” N15.*

This reduced the professionals’ confidence in using these models and posed significant challenges for administrators.

Additionally, experts reported that LLMs could pose serious violations of medical ethics. They thought LLMs might reflect societal biases in their training data, potentially leading to unfair outcomes in mental health assessments or recommendations.

*“they may provide inaccurate or discriminatory advice for certain demographic groups” N9.*

##### 3.2.2.2 Lack of professional nuance and depth

The next step after addressing misleading information should be to consider the lack of professional nuance and depth.

*“Currently, LLMs do not qualify as psychiatrists or mental health nurses, as they lack the specialized knowledge and core values essential for a professional in mental health” N18.*

Firstly, LLMs were considered to lack the depth of specialization required for mental health. They were designed to process and generate language based on patterns and data they had been trained on, but this did not equate to having a deep understanding of complex fields such as mental health.

Additionally, LLMs could provide information and some degree of support, but they lacked the ability for emotional understanding and expression. Consequently, in certain situations, LLMs might not offer support comparable to that of human professionals. In cases involving severe mental health issues or requiring emotional support, seeking assistance from trained clinical psychiatrists or nurses was still needed.

*“…LLMs cannot empathize with patients” N20.*

“In psychotherapy, there is a need for benevolent deception at times, which LLMs may not be able to accomplish, potentially even harming patients” N2.

##### 3.2.2.3 User risk

The flaws within the LLMs could be addressed through advancements in technology and technical improvements but the risks associated with users’ behaviors couldn’t be similarly resolved.

*“The shortcomings of LLMs in application in mental health like a ticking time bomb, healthcare professionals could inadvertently ignite this ‘bomb’ by misusing them”N18.*

Among all the risks, over-reliance and patient privacy breaches were the most frequently mentioned. Additionally, over-reliance on LLMs could potentially undermine their professional judgment leading to inappropriate medical decisions (N15). Patient privacy breaches pertained to the sensitivity and confidentiality of medical data. Inadequate processing or protection of this data could compromise patient privacy, potentially leading to legal ramifications and eroding trust (N5). Therefore, participants emphasized the importance of informed consent.

*“When using LLMs in mental health services, patients may not fully understand how their data is used, especially in model training and result generation. it is essential to explain the process and obtain patient consent” N14.*

*“Some patients may be unwilling to use such tools for therapy, their refusal must be respected.” N20.*

#### 3.2.3 Prerequisites for LLMs integration in mental health

##### 3.2.3.1 Training and competence

Providing training on LLMs knowledge for psychiatrists and mental health nurses could enhance the quality, efficiency, and safety of clinical practice(N17). Such training enabled professionals to better comprehend and apply this emerging technology, thereby delivering improved medical services and care to patients.

*“Conducting relevant training is essential for professionals to proficiently utilize LLMs and fully exploit their potential” N9.*

Additionally, participants emphasized that in this era of rapid AI advancement, enhancing digital literacy was a prerequisite for every healthcare professional (N7).

##### 3.2.3.2 Guidelines for use and management

In the mental health field, which involves sensitive information and human mental health, using LLMs rationally and safely is crucial. The lack of clear principles of usage and management might lead to confusion among healthcare when applying LLMs, thereby affecting their utilization and application in clinical practice (N16). Therefore, establishing clear guidelines and management mechanisms was essential to promote the widespread application of LLMs in mental health practice.

*“There is an urgent need for medical institutions or society to enact relevant regulations and usage protocols” N5.*

*“…It can constrain psychiatrists from abusing LLMs, preventing violations of medical ethics, as well as it can guide them in the use of LLMs” N11.*

##### 3.2.3.3 Patient engagement and transparency

The majority of participants considered informed consent from patients to be fundamental and necessary.

*“Obtaining explicit consent from patients is the foundation of respecting patient rights and medical ethics. N4.”*

Additionally, medical staff should explain to patients how they would use LLMs and the potential risks and limitations involved (N9). By ensuring patient involvement in the decision-making process and providing sufficient information, they could establish a foundation of trust and cooperation with patients, eventually, enhancing the effectiveness and quality of treatment.

#### 3.2.4 Expectations and future of LLMs in mental health

##### 3.2.4.1 Reasonable allocation of workload

Participants expressed their opinions and insights regarding the future role of LLMs in mental health, specifically focusing on the anticipated changes in the workload of psychiatrists and nurses and the ongoing development of these models.

The enhanced operational efficiency resulting from the implementation of LLMs should not equate to expectations of increased throughput (N20). Although the deployment of these models in mental health would incur additional healthcare costs, hospitals had to avoid excessively augmenting the workloads of psychiatrists and nurses purely for profit maximization, as this could compromise care quality and potentially precipitate patient safety incidents.

*“… hospitals will increase the workload of psychiatrists and nurses after integrating LLMs into clinical psychology” N9.*

“The efficiency improvements from LLMs could potentially become a burden for healthcare workers, meaning they might need to treat more patients in the same amount of time.”

##### 3.2.4.2 Upgrades and revamps of LLMs

Upgrades and revamps of LLMs are essential for their application in clinical psychology in the future. To this end, experts expected technology companies could develop a version of LLMs specifically tailored for mental health practice.

*“To date, few LLMs have had pre-training with the corpus of clinical psychology or with millions of mental illness records, images, lab data…” N16.*

LLMs tailored for mental health practice required systematic and scientific corpus training. The information generated should also be verified by a review mechanism to ensure its usability (N8).

## 4 Discussion

In this study, we explored mental health experts’ perceptions of integrating LLMs into mental health practice. Participants discussed the significant transformations brought by LLMs into mental health practice (fair access to mental health services, enhancement of patient participation, improvement in work efficiency and quality) and analyzed the current gaps in their application within mental health (misleading information, lack of professional nuance and depth, user risk). Based on these points, they provided a range of possible ways in which LLMs could be embedded within mental health (training and competence, guidelines for use and management, patient engagement and transparency) and expressed their expectations for future developments (reasonable allocation of workload, upgrades and revamps of LLMs). The research findings provide valuable insights into the integration of LLMs in mental health practice, as well as offer significant references for conducting and implementing related studies.

Similar to much of the existing literature on the perspectives of AI for mental health (18, 19), respondents were excited about the potential benefits of LLMs in mental health. LLMs encompass significant changes affecting both mental health professionals and patients. For psychiatrists and mental health nurses, the reform aims to improve the efficiency and quality of work. Notably, this study also emphasized the potential of LLMs to enhance public access to mental health services and increase patient participation in psychotherapy, which supplemented the research by Zhang et al. (20) and Miner et al. (21). Previous literature similarly pointed out that before the advent of LLMs like ChatGPT, related chatbots had been developed for mental health treatment. They were available anytime and could help those uncomfortable with seeing a therapist or people with limited access to mental health services (18, 22). However, a systematic review indicated that previous implementations of chatbots for mental health interventions might have performed inadequately when addressing complex tasks. These chatbots failed to effectively identify and respond to patients’ emotions and needs, leading to diminished patient confidence and consequently impacting overall treatment outcomes (23). This decline in trust further restricted the widespread adoption and effectiveness of chatbots in clinical applications. However, LLMs hold significant potential to address these challenges in the future (24). They could markedly enhance emotional understanding and empathy, enabling more nuanced interpretations and responses to users’ emotional needs. Furthermore, LLMs demonstrate advanced capabilities in managing complex dialogues and situations, dynamically adjusting their responses to more effectively support intricate mental health issues (3).

Like most AI products used in mental health practice, LLMs face various challenges when applied in this area and cannot fully replace professionals, especially in ethical judgment and managing patient privacy (25). These challenges stem from both user-related risks and inherent limitations of the LLMs themselves. Our study found that user-related risks included privacy and confidentiality. Professionals may inadvertently disclose patient information during the diagnostic and treatment processes, potentially leading to data security issues if not properly managed, it is similar to the finding of Demszky et al. (6). Therefore, it is recommended that healthcare systems should develop their own LLMs rather than rely on external providers like OpenAI. This approach can help mitigate privacy-related concerns among professionals and enhance the effectiveness of clinical applications. There is also the risk of users developing an over-reliance on LLM-generated responses, which may result in neglecting professional advice or misinterpreting guidance without appropriate context. Inherent limitations of LLMs involve biases present in training data that can lead to inaccurate or inappropriate responses, especially in diverse populations with varying cultural backgrounds. Several studies have indicated that the existing research literature on mental health largely represents the perspectives of people who are educated, are of high socioeconomic status, and speak English (25, 26). Consequently, LLMs, trained on such data, may lack sufficient representation of diverse cultural, socioeconomic, and linguistic backgrounds. Additionally, LLMs lack genuine empathy and the ability to recognize nuanced emotional cues, which are crucial in mental health interventions and they may fail to identify crises that require immediate human intervention, potentially delaying critical support. For example, a study has demonstrated that chatbots do not consistently or adequately respond to suicide risk, at times being dismissive and neglecting to provide crisis resources or referrals to human providers (27). Therefore, mental health-related professionals should foster a pragmatic attitude and exercise vigilance towards professional content generated by LLMs.

However, some experts reported that merely being vigilant with LLMs was far from sufficient, which is similar to Julia et al. (28). They emphasized the limits of clinician vigilance as an AI safety bulwark. In this viewpoint, participants acknowledged the intricate reality and the existing gap between the technology and mental health fields and proposed pertinent prerequisites that primarily included three key stakeholder groups. For frontline mental health doctors and nurses, digital literacy is essential for effectively utilizing such AI products. A study has found that medical staff may be uncomfortable with AI integration because of low digital literacy, ultimately leading to changes in their usage attitudes and intentions (29). Especially for LLMs like ChatGPT, which have not been released for a long time, and with continuous updates and revisions, professionals should receive related training and support. For healthcare institutions, guidelines for use and management are urgent. This approach may appear to constrain their behavior, but it primarily aims to guide their use of technologies. It even offers protection by providing a clear framework for managing sensitive data and navigating ethical dilemmas. For patients, it is imperative to be informed when LLMs are used in mental health treatment, as this upholds their right to informed consent. However, some participants indicated that informing patients about the use of LLMs in their healthcare could potentially hinder the implementation of these technologies. This viewpoint has been confirmed by previous studies, one key concern revolves around patient acceptance and comfort with AI (30). While many patients recognize the potential benefits of AI in healthcare, such as improved diagnostic accuracy, there remains a significant level of discomfort and skepticism about replacing human judgment with AI solutions. This skepticism may stem from a lack of understanding of how AI works, fears about privacy and data security, and concerns about the loss of personal interaction with healthcare providers (20). Therefore, we can through effective communication and education enhance patients’ understanding and trust in this technology. For instance, clearly demonstrating to patients how the technology can improve treatment outcomes. Eventually, providing patients with the option to choose whether to use this new technology respects their autonomy.

The prerequisites for integrating LLMs into the field of mental health are foundational steps and the subsequent discussion on “expectations” is the key step for LLMs to be continuously applied in the mental health field. As users of LLMs, mental health doctors and nurses hope hospitals can reasonably allocate work based on actual conditions. This aligns with the findings of Petersson et al. (30), in which healthcare workers may worry that hospital management will expect significant improvements in work efficiency and quality after introducing AI technology. Such expectations can add extra pressure and increase their workload, ultimately becoming an obstacle to the implementation of LLMs in practical work. Therefore, management should allocate work based on actual conditions rather than raising unrealistic expectations for efficiency and quality. Firstly, continuous monitoring and adjustment of workflows are essential to ensure LLMs serve as an aid rather than increase the workload. Subsequently, transparent and open communication with professionals is crucial to ensure the effective practical application of LLMs. Finally, providing adequate support and resources, including technical assistance and psychological support, is necessary during the transition to LLM integration (31). The content generated by LLMs is based on continuously evolving data. Additionally, to ensure the timeliness and reliability of these models in providing mental health-related information, regular updates and revisions are necessary to incorporate new information and correct biases. This ongoing process is crucial to ensure that the models can continually meet the changing needs of users. Based on the findings of this study and our previously published scoping review, five members of our research team conducted a group discussion to develop a guideline for using LLMs in mental health practice, as shown in Supplementary material S3. Future research should focus on advancing the application of LLMs in mental health by exploring several key areas. Conducting mixed-method studies that integrate qualitative and quantitative approaches will provide comprehensive insights into mental health professionals’ experiences with LLMs and the factors influencing their use. Developing specialized assessment tools specific to LLMs in mental health will enable a scientific evaluation of their effectiveness within this domain. Additionally, implementing long-term, multicenter, large-sample, high-quality randomized controlled trials will assess the specific efficacy of these models in mental health, thereby informing the development of more effective clinical applications.

### 4.1 Strengths and limitations

This study contributes to a significant research gap regarding the expectations of mental health nurses, psychiatrists, and computer scientists on the development and implementation of LLMs. These professionals highlighted the potential of LLMs to improve diagnostic accuracy, develop personalized treatment plans, and enhance access to mental health services. Additionally, the research findings provide valuable insights into the integration of LLMs into the field of mental health, offering significant references for conducting and implementing related studies, which lay a solid foundation for future applications and studies.

Despite these strengths, the interpretation of our findings may be limited by the study being conducted in just four provinces in China. Additionally, a notable limitation of this study is the potential for self-selection bias as participation was voluntary, and the expert-based interview approach may result in a sample that does not fully represent the broader mental health professionals, particularly those who may be less familiar with LLMs.

## 5 Conclusion and recommendations

This study provided valuable insights into the potential and challenges of integrating LLMs into mental health practice from the perspectives of experts. The findings indicated that LLMs could significantly reform practice and application by improving access to mental health services, enhancing patient participation, and increasing work efficiency and quality. However, there were substantial technological-mental health gaps, including the risk of disseminating misleading information, a lack of professional nuance, and potential user risks. To address these issues, experts highlighted the necessity of comprehensive training and competence development, the establishment of robust guidelines for use and management, and ensuring patient engagement and transparency. Looking ahead, participants anticipated a balanced allocation of workload and ongoing advancements and refinements in LLMs. This study underscored the importance of meticulous planning and structured implementation to maximize the benefits while mitigating the risks associated with integrating LLMs into mental health practice.

## Data Availability

The original contributions presented in the study are included in the article/Supplementary material, further inquiries can be directed to the corresponding authors.
