# Supplementary material for: Integrating large language models in mental health practice: a qualitative descriptive study based on expert interviews
Source: Front Public Health. 2024 Nov 4;12:1475867. doi: 10.3389/fpubh.2024.1475867 (PMC11571062; doi:10.3389/fpubh.2024.1475867)
Supplement: Supplementary file 2 [file Data_Sheet_2.docx]

**Integrating large language models in mental health: a qualitative descriptive study based on expert interviews**

**一、Introduction**

Q1. How did you learn about the Large Language Models?

Q2. Which large language model are you primarily using right now?

Q3. Please recall and describe an impressive event from the last one or two uses.

**二、Formal interview outline**

Q1. What is your current attitude towards using large language models in mental health field?

Q2. Please share your specific views on the use of large language models in mental health field.

Q3. What do you think are the potentials and risks of using large language models in mental health field?

Q4. Please share your specific perspectives on the possibilities, conditions and prerequisites of large language models integration in pathology are explicated

Q5. What’s your expectation or advice about using large language models in mental health field?
