# Supplementary material for: Integrating large language models in mental health practice: a qualitative descriptive study based on expert interviews
Source: Front Public Health. 2024 Nov 4;12:1475867. doi: 10.3389/fpubh.2024.1475867 (PMC11571062; doi:10.3389/fpubh.2024.1475867)
Supplement: Supplementary file 3 [file Data_Sheet_3.docx]

| **Guidelines for Use** | |
| --- | --- |
| **Items** | **details** |
| **Informed consent** | Patients must be informed about the specific applications of large language models (LLMs) in their treatment, including the stages at which they are utilized, the objectives, and the potential risks and benefits |
| **Privacy & Data Management** | **Data Anonymization**: Utilize pseudonyms or other advanced anonymization techniques to protect patient identity information and mitigate the risk of data breaches. |
|  | **Access Control**: Implement stringent access control measures to ensure that only authorized personnel can access sensitive data. |
|  | **Regular Cheak**: Conduct regular data security checks to identify potential security vulnerabilities. |
| **Basic Support** | **Feedback Mechanism**: Establish an effective feedback mechanism to collect input from mental health professionals and patients, thereby continuously optimizing the use of LLMs. |
|  | **Maintenance Mechanism:** Develop a comprehensive emergency response plan to address malfunctions promptly and effectively, ensuring minimal disruption to clinical services. |
|  | **Financial Support:** seek governmental or institutional grants, and establish partnerships with industry stakeholders. Additionally, implement phased investments to distribute financial burdens over time, ensuring sustainability without compromising the quality of service. |
| **Implementation Strategies** | **Appropriate Selection**: Enable patients to choose LLMs and corresponding services at various pricing tiers based on their needs and preferences. |
|  | **System Integration:** Integrate LLMs with existing mature clinical information systems to ensure seamless data transmission and sharing. |
|  | **Phased Implementation:** Adopt a phased implementation strategy by piloting the application in select departments or clinical workflows before broader deployment. |
|  | **Establish a Review Process:** Clearly define the number of mental health professionals required to sign and validate the content generated by large language models (LLMs) before it can be adopted. |
| **Training and capacity building** | **Multidisciplinary Training:** Engage experts from multiple disciplines (Artificial Intelligence, Medicine, Nursing, Psychology, etc.) to design scientifically rigorous training programs. |
|  | **Diversified Training Methods:** Consider the specific work contexts of mental health professionals and implement a combination of online conferences and in-person lectures. |
|  | **Comprehensive Content:** Ensure training content is specific and thorough, encompassing technical fundamentals, operational guidelines, and ethical standards. |
|  | **Establish Evaluation Criteria:** Conduct knowledge and skill assessments post-training, granting usage qualifications only to professionals with a “good” rating or higher. |
